# Socio-professional stigmatization among healthcare workers with COVID-19

**DOI:** 10.1192/j.eurpsy.2023.1714

**Published:** 2023-07-19

**Authors:** M. N. Fendri, D. Brahim, N. Mechergui, I. Youssef, S. Ernez, H. Ben Said, N. Ladhari

**Affiliations:** Occupational disease and fitness for work, Charles Nicolle Hospital, Tunis, Tunisia

## Abstract

**Introduction:**

The COVID-19 pandemic had deeply altered the social and professional lives of people with SARS-COV2. The anxiety of being contaminated by the virus during the first waves had created avoidance behaviors and established a climate of rejection towards healthcare workers (HW) with COVID.

**Objectives:**

The aim of this study was to assess stigmatization among healthcare workers with COVID-19

**Methods:**

This is a retrospective cross-sectional study carried out on HWs in a university hospital in Tunis who were affected by COVID-19 and who consulted the occupational medicine department. The study was conducted between March 2021 and June 2021. Data collection was based on pre-established forms. The questionnaire assessing stigmatization was inspired by the questionnaire assessing stigma in AIDS patients

**Results:**

The study included 100 health personnel. The sex ratio (M/W) = 0.29. The average age was 39.22 ± 9.3 with extremes ranging from 24 to 58 years. The average professional seniority was 11.39±9.4. Nurses were the most represented professional category (26%). The psychiatric history was: Depressive disorder (14%) and anxiety disorder (10%). Eighty HW were infected with SARS-COV2 for the first time. Contamination was intra-hospital in 50% of cases. Eighteen HW had been rejected. Verbal abuse towards HW with COVID was noted in 8% and physical abuse in 11%. Twenty-six HW had lost their friends and 36 of them no longer had as much social activity as before. In the workplace, rejection was noted in 21% cases, 10 health personnel reported a discriminatory orientation for the care of patients with COVID and 19 HW felt useless at work.

**Conclusions:**

Socio-professional stigmatization should help us to understand the vulnerability and psychological impact of this health crisis on health workers. Control and prevention strategies need to be established.

**Disclosure of Interest:**

None Declared

